# Mapping sleep disturbance and nap desire to dimensions of psychopathology in U.S. adults

**DOI:** 10.3389/fpsyg.2026.1828219

**Published:** 2026-09-03

**Authors:** Amelia M. Stanton, Devisi A. Ashar, Martin Seehuus

**Affiliations:** 1 Department of Psychological and Brain Sciences, Boston University, Boston, MA, United States; 2 Middlebury College, Middlebury, VT, United States; 3 Vermont Psychological Services, University of Vermont, Burlington, VT, United States

**Keywords:** insomnia, nap desire, napping, psychopathology, sleep disturbance, transdiagnostic analysis

## Abstract

**Study objectives:**

Insomnia and nap desire are linked to heightened psychopathology, yet less is known about their associations with broad psychopathology dimensions. Wright and colleagues recently developed brief transdiagnostic scales assessing internalizing, detachment, antagonism, disinhibition, and anankastia, but these have not been evaluated in relation to sleep. We examined cross-sectional psychopathology profiles in relation to probable insomnia and daily nap desire in U.S. adults.

**Methods:**

Participants (*N* = 831; analytic *n* = 705 after attention checks) were recruited via Prolific.com and stratified into clinical (receiving psychological/psychiatric treatment) and non-clinical groups. Measures included the PHQ-9 (depression), GAD-7 (anxiety), Sleep Condition Indicator (SCI), five-factor psychopathology scales, and nap desire/behavior. Probable insomnia was defined using the SCI clinical cut-off. Analyses tested differences in psychopathology dimensions by insomnia and nap desire.

**Results:**

Participants with probable insomnia showed significantly different psychopathology profiles than those without, *F*(1,703) = 178.57, *p* < 0.001, partial η^2^ = 0.20, with elevated internalizing, detachment, disinhibition, and antagonism, but not anankastia. Nap desire was associated with higher psychopathology across dimensions, though effects were smaller, *F*(1,703) = 36.78, *p* < 0.001, partial η^2^ = 0.05.

**Conclusion:**

Probable insomnia and daily nap desire corresponded to elevated psychopathology profiles, with stronger, more differentiated effects for insomnia. Dimensional frameworks may inform sleep-focused and transdiagnostic interventions, particularly targeting internalizing and detachment.

## Introduction

Sleep disturbance is common across a range of mental health conditions and is both a symptom and risk factor for psychopathology (Harvey et al., 2011; Riemann et al., 2020; Guerrera et al., 2025). Insomnia has robust associations with mood, anxiety, and substance use disorders, and is increasingly recognized as a potential transdiagnostic factor that can exacerbate or maintain psychiatric symptoms (Harvey, 2008; Pigeon et al., 2017). Emerging neurobiological models conceptualize insomnia as a disorder of persistent hyperarousal involving dysregulation of arousal-promoting systems, particularly the orexin (hypocretin) system. Contemporary reviews suggest that these mechanisms could contribute to both the development and maintenance of chronic insomnia and help explain the disorder’s broad associations with emotional dysregulation and psychopathology, while also informing the development of targeted pharmacologic and behavioral treatments (Mogavero et al., 2023). This biological framework complements behavioral and transdiagnostic conceptualizations of insomnia by providing plausible mechanistic pathways through which sleep disturbance affects mental health. Meta-analytic and longitudinal evidence indicates that insomnia prospectively predicts depression, anxiety, and substance use disorders, even after controlling for baseline symptoms (Baglioni et al., 2011; Hertenstein et al., 2019).

Excessive daytime sleepiness and daytime napping are also linked to mental health problems, including depression, though their relationships appear to be more variable and may depend on contextual or individual factors (Hayley et al., 2013; Hein et al., 2019; Li et al., 2022; Ohayon, 2008). Recent work suggests that nap desire (i.e., wanting to nap regardless of opportunity), which may be driven by excessive daytime sleepiness in some individuals (though nap desire is likely influenced by broader affective, motivational, and contextual processes), may be more closely related to psychological distress than nap behavior itself (Ostrow et al., 2024). This suggests that wanting to nap may reflect an avoidant or dysregulated process, while actually napping may be more a function of opportunity.

Because relationships between sleep disturbance and psychopathology are complex and bidirectional, identifying their underlying pathways has proved challenging (Akpoveta et al., 2025; Li et al., 2022). Prior research has linked insomnia to both categorical diagnoses and dimensional traits, including negative affectivity, anhedonia, hostility, impulsivity, and perfectionism (Harvey, 2002; Bonnet and Arand, 2010; Taylor et al., 2013; Kamphuis et al., 2014; Schmidt et al., 2010; Schmidt et al., 2008; Jansson-Fröjmark and Linton, 2007). For example, insomnia severity is strongly associated with internalizing-spectrum traits such as negative affectivity and neuroticism (Meneo et al., 2023; Baglioni et al., 2010; van de Laar et al., 2010), while sleep disturbance has also been linked to detachment-related processes, including social withdrawal and reduced reward sensitivity (Ben Simon and Walker, 2018; Goldstein and Walker, 2014). Disinhibition-related traits such as impulsivity have been associated with irregular sleep patterns and poor sleep hygiene (McGowan and Coogan, 2018; Panjeh et al., 2025), and antagonism and hostility have been linked to both short sleep duration and insomnia complaints (Grano et al., 2008). Finally, compulsivity and perfectionism have also been implicated in pre-sleep cognitive arousal and insomnia maintenance (Nota et al., 2015; Küskens et al., 2024; Schmidt et al., 2018; Stricker et al., 2022). Consistent with the work of the HiTOP consortium, dimensional frameworks offer substantial insights into understanding comorbidity and transdiagnostic processes related to sleep (Kotov et al., 2017; Komasi et al., 2025; Phillips et al., 2025).

More recent work has similarly emphasized that sleep disturbances and circadian rhythm disruption should be considered within broader transdiagnostic frameworks rather than solely as symptoms of individual psychiatric disorders. Reviews have highlighted that disrupted sleep and biological rhythms are common across multiple forms of psychopathology and are associated with impairments in emotional, cognitive, and psychosocial functioning (Harvey et al., 2011; Walker et al., 2020). Recent empirical work further demonstrated that disturbances in biological rhythms and sleep quality were associated with poorer psychosocial functioning among individuals with major depressive disorder, with biological rhythm disruption partially accounting for the association between depressive symptom severity and functional impairment (Guerrera et al., 2025). Collectively, these findings support examining sleep-related experiences within dimensional models of psychopathology that cut across traditional diagnostic boundaries.

While emerging evidence suggests that sleep disturbances cut across multiple higher-order psychopathology dimensions, most studies have examined these traits in isolation or within traditional diagnostic frameworks. Dimensional models such as HiTOP provide a structured, hierarchical framework for organizing these traits and examining whether sleep disturbances map onto specific higher-order spectra (e.g., internalizing, detachment, disinhibition) rather than discrete disorders. By situating sleep-related phenomena within this broader structure, a dimensional approach may clarify whether insomnia and nap desire show distinct psychopathology “profiles” across spectra, which could inform more targeted intervention strategies.

In 2025, Wright and colleagues developed a set of brief transdiagnostic scales designed to capture broad domains of psychological functioning that cut across traditional diagnostic categories (Wright et al., 2025). These measures assess five key dimensions: *internalizing* (i.e., negative affectivity, anxiety, depression), *detachment* (i.e., social withdrawal, anhedonia), *antagonism* (i.e., hostility, oppositionality), *disinhibition* (i.e., impulsivity, poor self-control), and *anankastia* (i.e., rigidity, perfectionism, compulsivity). Although each spectrum is assessed with a small number of items, these scales were developed using item-response modeling to maximize coverage of core spectrum variance while minimizing participant burden. Their brevity makes them well-suited for large-scale screening studies and exploratory mapping of constructs across domains, though they are not intended to replace full-spectrum measures like the HiTOPS measures themselves.

Understanding the dimensional psychopathological correlates of sleep disturbances has important clinical implications. Insomnia has been consistently associated with heightened negative affect, avoidance, and impaired emotion regulation, suggesting that interventions targeting these domains may reduce both sleep problems and broader psychiatric symptoms (Harvey, 2022; Swift et al., 2022). Daytime sleepiness and the desire to nap, while less studied, may similarly signal a desire to avoid daily hassles, and thus may reflect affective dysregulation. Thus, nap desire may provide a potential marker of risk for emerging psychopathology (Li et al., 2022; Ostrow et al., 2024; Ohayon et al., 1997). By examining how insomnia and nap desire align with psychopathology dimensions, this work may help identify transdiagnostic targets for future intervention development.

Given the existing literature linking sleep disturbance to dimensional models of psychopathology, several hypotheses emerge, described below. However, given the limited (although compelling) work supporting the transdiagnostic measures used (Wright et al., 2025), analyses involving the scales developed by Wright et al. (2025) should be understood as exploratory in nature.

First, we expected to replicate and expand existing findings, such that (H_1_) participants who reported currently receiving mental health care would show more severe insomnia symptoms, greater fear of sleep, higher scores on the transdiagnostic and mental health scales, and greater nap desire. Further, given that insomnia has repeatedly been associated with heightened negative emotionality, worry, and rumination (Baglioni et al., 2011; Hertenstein et al., 2019; Harvey, 2002; Bonnet and Arand, 2010), suggesting a particularly strong alignment with the internalizing domain, we hypothesized that (H_2_) participants with probable insomnia would demonstrate significantly elevated internalizing-spectrum scores relative to those without insomnia. Poor sleep has been implicated in diminished social engagement and blunted positive affect (Ben Simon and Walker, 2018; Goldstein and Walker, 2014; Franzen and Buysse, 2008), which are central features of detachment-related constructs. Given those findings and other work linking chronic sleep disturbance to anhedonia, social withdrawal, and reduced reward sensitivity, we expected (H_3_) insomnia to also show positive associations with detachment.

Since cognitive models of insomnia highlight pre-sleep rumination, rigid sleep-related beliefs, and perfectionistic tendencies as maintaining factors, suggesting theoretical convergence with anankastia-related traits (Jansson-Fröjmark and Linton, 2007; Johann et al., 2023), we anticipated (H_4_) modest positive associations between insomnia and anankastia (rigidity, perfectionism, compulsivity). Although hostility and short sleep duration have been linked in some studies, findings are less consistent relative to other spectra (Kamphuis et al., 2012; Ireland and Culpin, 2006). Thus, associations between sleep variables and antagonism were considered exploratory, and we examined antagonism without a strong directional hypothesis.

Given the comparatively limited literature on nap desire specifically, hypotheses involving nap desire were informed by theoretical models of avoidance and dysregulation but are also exploratory.

Finally, we hypothesized that profile analyses of (H_5_) probable insomnia and (H_6_) nap desire would demonstrate different patterns of dimensions of psychopathology, reflecting the different complex associations between sleep and psychopathology. These profile differences are useful, if observed, because they support existing evidence that the connections between sleep disruption and psychopathology are multifactorial and complex.

It is important to note that the present study is cross-sectional, and thus these hypotheses concern dimensional associations rather than causal mechanisms. This cross-sectional design does not test causal pathways or mechanisms; rather, it evaluates whether distinct dimensional profiles are observable across sleep-related experiences.

Therefore, the current study leverages Wright et al.’s (2025) brief transdiagnostic scales to systematically examine how insomnia and nap desire correspond to broad psychopathology dimensions in both clinical and non-clinical samples. By comparing people with and without probable insomnia, and those with differing levels of nap desire, we evaluate whether distinct psychopathology signatures are associated with each sleep-related experience. In doing so, this study extends prior work linking sleep to individual traits by situating these associations within a hierarchical dimensional framework and providing preliminary evidence that may help guide future longitudinal and intervention research examining spectrum-specific associations.

## Methods

The study was approved by the senior author’s Institutional Review Board (IRB Protocol #468) prior to the start of the study. Participants (*N* = 831) were recruited through Prolific.com (Prolific, 2020) and were eligible if they were over 18 years old and located in the United States. All participants provided informed consent prior to participation. Data were collected through Qualtrics (2025), and participant recruitment and compensation were conducted through Prolific.

### Participants

Three attention check questions were used. Based on the work of Curran and Hauser (2019), two questions were selected from existing work that demonstrated very low false-positive rates: “I have been to every country in the world” (failed by *n* = 26) and “I work fourteen months in a year” (failed by *n* = 75) (Huang et al., 2015; Meade and Craig, 2012). An additional, similarly implausible question was added to be thematically consistent with the sleep measure in which it was embedded (“I sleep on a pillow made of spaghetti,” failed by *n* = 92). There was considerable overlap in the participants failing each of the questions, with *n* = 705 passing all three and *n* = 126 failing at least one. Excluded participants were somewhat younger (M = 37.2 vs. 40.0, *d* = 0.21), higher in subjective SES (*d* = −0.44), and more likely to be in the clinical group (62.7% vs. 47.5%, *φ* = 0.11); they also differed in sexual orientation (excluded more likely to identify as gay or lesbian; Cramér’s V = 0.17) and racial composition (excluded participants were more likely to identify as Black; V = 0.13), though several cells had low expected counts. Gender did not differ (V = 0.04). The differences were small to medium in effect size. Out of caution, all analyses were conducted on the full sample and the most restricted sample, with no changes in significance or sign of the observed effects. For that reason, all analyses described below represent the most restricted sample, *n* = 705.

Half of the sample was restricted to participants who reported that they were not currently receiving psychological or psychiatric treatment (non-clinical, *n* = 370 who passed the attention checks), and half to participants who reported currently receiving psychological or psychiatric treatment (clinical, *n* = 335 who passed the attention checks). We did not capture and cannot confirm the presence of a diagnosis, and thus the distinction between clinical and non-clinical should be understood solely in terms of participants’ self-reported receipt of psychological treatment. See Table 1 for demographics based on clinical vs. non-clinical samples.

**Table 1:** Demographics and variables of interest by clinical vs. non-clinical sample.

Variable		Non-clinical	Clinical	All		
	Non-clinical vs. clinical	M ± SD/*n* (%)	M ± SD/*n* (%)	M ± SD/*n* (%)	Effect size	
					Point est.	95% CI
Gender	*χ*^2^(2) = 3.84, *p* = 0.15				*v* = 0.07	
Man		174 (47.03%)	146 (43.58%)	320 (45.39%)		
Woman		190 (51.35%)	176 (52.54%)	366 (51.91%)		
TGD		6 (1.62%)	13 (3.88%)	19 (2.70%)		
Sexual orientation	*χ*^2^(3) = 42.77, *p* < 0.001				*v* = 0.25	
Straight		318 (85.95%)	218 (65.07%)	536 (76.03%)		
Bi		35 (9.46%)	71 (21.19%)	106 (15.04%)		
Gay		10 (2.70%)	24 (7.16%)	34 (4.82%)		
Other		7 (1.89%)	22 (6.57%)	29 (4.11%)		
Age	*t*(702.29) = 0.75, *p* = 0.45	40.38 ± 13.93	39.64 ± 12.21	40.03 ± 13.14	*d* = 0.06	−0.09, 0.20
SES	*t*(703) = 2.26, *p* = 0.02	5.42 ± 1.60	5.13 ± 1.75	5.28 ± 1.68	*d* = 0.17	0.02, 0.32
Mental health						
Anxiety (GAD-7)	*t*(653.89) = −11.51, *p* < 0.001	3.99 ± 4.82	8.63 ± 5.76	6.20 ± 5.77	*d* = −0.88	−1.03, −0.72
Depression (PHQ-9)	*t*(636.52) = −11.74, *p* < 0.001	4.70 ± 5.32	10.09 ± 6.70	7.26 ± 6.58	*d* = −0.90	−1.05, −0.74
Wright scales						
Internalizing	*t*(660.18) = −10.544, *p* < 0.001	1.66 ± 1.98	3.38 ± 2.32	2.48 ± 2.31	*d* = −0.80	−0.96, −0.65
Detachment	*t*(639.25) = −11.02, *p* < 0.001	1.66 ± 2.10	3.65 ± 2.62	2.61 ± 2.56	*d* = −0.84	−0.99, −0.69
Antagonism	*t*(585.96) = −4.49, *p* < 0.001	0.62 ± 1.27	1.16 ± 1.84	0.88 ± 1.59	*d* = −0.34	−0.49, −0.20
Disinhibition	*t*(667.31) = −5.27, *p* < 0.001	1.74 ± 1.83	2.52 ± 2.09	2.11 ± 2.00	*d* = −0.40	−0.55, −0.25
Anankastia	*t*(697.20) = 0.38, *p* = 0.71	3.19 ± 2.46	3.13 ± 2.44	3.18 ± 2.45	*d* = 0.03	−0.12, 0.18
Sleep						
Sleep condition (SCI)	*t*(703) = 10.61, *p* < 0.001	21.55 ± 7.77	15.30 ± 7.85	18.58 ± 8.41	*d* = 0.80	0.65, 0.95
Fear of sleep (FoSI-SF)	*t*(571.27) = −8.23, *p* < 0.001	5.95 ± 8.46	12.73 ± 12.74	9.17 ± 11.22	*d* = −0.63	−0.78, −0.48
Napping frequency	*F*(1, 703) = 0.42, *p* = 0.52					
Never		38 (10.27%)	41 (12.24%)	79 (11.21%)		
Rarely		91 (24.59%)	80 (23.88%)	171 (24.26%)		
1–2/month		56 (15.14%)	32 (9.55%)	88 (12.48%)		
1–2/week		96 (25.95%)	88 (26.27%)	184 (26.10%)		
Most days		68 (18.38%)	68 (20.30%)	136 (19.29%)		
Every day		21 (5.68%)	26 (7.76%)	47 (6.67%)		
Nap desire	*F*(1, 703) = 7.34, *p* = 0.007					
Yes		170 (45.95%)	188 (56.12%)	358 (50.78%)		
No		200 (54.05%)	147 (43.88%)	347 (49.22%)		
Nap actual	*F*(1, 703) = 0.70, *p* = 0.41					
Yes		87 (23.51%)	70 (20.90%)	157 (22.27%)		
No		283 (76.49%)	265 (79.10%)	548 (77.73%)		

*T*-tests with fractional degrees of freedom had significant Levene’s test values.

In this table, demographic, mental health, psychopathology dimensions, and sleep-related characteristics by clinical vs. non-clinical groups.

### Measures and procedure

Participants were asked basic demographic information, including age, race, ethnicity, gender, and subjective socioeconomic status (SES). In addition, participants completed measures of depression [PHQ-9 (Kroenke et al., 2001)], anxiety [GAD-7 (Spitzer et al., 2006)], sleep condition [SCI (Espie et al., 2014)], the abbreviated scales assessing the structure of psychopathology (Wright et al., 2025), and brief questions about napping (Ostrow et al., 2024).

#### Depressive symptoms (PHQ-9)

The PHQ-9 (Kroenke et al., 2001) consists of nine Likert-style multiple-choice items that probe the frequency of depressive symptoms in the past 2 weeks. Symptoms include “Little interest or pleasure in doing things” and “Poor appetite or overeating,” with options varying from not at all (scored zero) to nearly every day (three). Scores thus range from zero to 27, with commonly used interpretive breakpoints at five (less than five is minimal depression), 10, 15, and 20 (more than 20 is severe depression) (Kroenke et al., 2001). The last question assesses both passive and active suicidality (“Thoughts that you would be better off dead or of hurting yourself in some way.”) The PHQ-9 has demonstrated strong internal reliability (*α* = 0.89), which was also observed in this sample (α = 0.92).

#### Anxiety symptoms (GAD-7)

The GAD-7 (Spitzer et al., 2006) consists of seven Likert-style multiple-choice items that assess the frequency of anxiety symptoms in the past 2 weeks. Symptoms include “Trouble relaxing” and “Worrying too much about different things,” with options ranging from not at all (scores zero) to nearly every day (three). Scores thus range from zero to 21, with commonly used interpretive breakpoints at five (less than five is indicative of minimal anxiety), 10, and 15 (more than 15 suggests severe anxiety). The GAD-7 has demonstrated strong internal reliability (*α* = 0.92) (Spitzer et al., 2006), which was also observed in this sample (α = 0.94).

#### Sleep Condition Indicator (SCI)

The Sleep Condition Indicator (Espie et al., 2014) includes eight Likert-style multiple-choice items about sleep, including “Thinking about a typical night in the past month, how long does it take you to fall asleep?” and “Thinking about the past month, to what extent has poor sleep troubled you in general?” (Espie et al., 2014) Higher scores on the SCI represent better sleep quality, such that an answer of “zero to fifteen minutes” for “How long does it take you to fall asleep?” would be scored a four, while “more than 61 min” would be scored a zero. This scoring system distinguishes the SCI from similar measures, such as the Insomnia Severity Index (Morin et al., 2011), for which higher scores represent more insomnia. The SCI has an established cut-off score of <16 as suggestive of clinically meaningful insomnia (Espie et al., 2014; Bayard et al., 2021; Sheaves et al., 2016), which we used here. The SCI demonstrated strong internal reliability (*α* = 0.86) (Espie et al., 2014), which was also observed in this sample (α = 0.94).

#### Dimensions of psychopathology

The subset of the scales described in Wright et al. (2025) used here was developed to track within-person variability in basic dimensions and processes underlying psychopathology. That is, it was *not* developed for use as a cross-sectional measure of psychopathology, although the underlying approach(es) toward reconceptualizing psychopathology [described by others (Kotov et al., 2017; Caspi et al., 2024; Forbes et al., 2024; Ringwald et al., 2023)] has tended toward cross-sectional work. Thus, our use of this brief 15-item five-factor scale represents an extension of the work of Wright et al. (2025), and not a simple application of it. The scale we used in this study was identified by Wright et al. (2025) as an example of their method of developing brief measures for daily domain assessments. It includes items like “I felt tense” (from the internalizing subscale) and “I made sure everything I did was perfect” (from the anankastia subscale). Items were scored from zero (not at all true for me) to three (usually or almost always true for me). Since there were three items in each subscale, scores within each subscale ranged from zero to nine. Observed internal reliability was adequate, with *α* varying from a low of 0.72 for Disinhibition to 0.77 for Anankastia, followed by 0.79 for Antagonism, 0.79 for Internalizing, and 0.84 for Detachment. The brevity of the measure (three items per construct) is a strength and a weakness. While participants are likely to maintain attention through such a brief measure, a three-item scale has inherent limitations in terms of range and measurement challenges in assessing internal reliability.

#### Fear of sleep (FoSI-SF)

Fear of sleep was measured with the Fear of Sleep Inventory––Short Form (FoSI-SF) (Pruiksma et al., 2014), a 13-item, five-level, Likert-style scale intended to capture fear of sleep (Zayfert et al., 2006). This questionnaire includes items like “Being in the dark scared me” and “I felt that it was dangerous to close my eyes,” with options from 0 = “Not at all” to 4 = “Nearly every night.” Higher scores thus reflect more fear of sleep. The FoSI-SF demonstrated strong internal reliability in the validation study (*α* = 0.90) and in this sample (α = 0.95).

#### Napping

A brief set of questions about napping desire (“Did you want to take a nap today?”) and napping behavior (“Did you take a nap today?” and “How often do you take naps, generally speaking?”) was included. The distinction between napping desire and napping behavior is based on recent work (Ostrow et al., 2024) that suggests that napping desire is more strongly associated with distress and psychopathology than napping behavior, with the authors speculating that napping behavior is likely inhibited or enabled by externalities like employment, school, and family responsibilities. Participants were asked how frequently they usually napped (from never to daily), if they napped today, and if they wanted to nap today. If they indicated that they wanted to nap but did not, they were asked to identify the reasons they did not nap, from work obligations to sleep disruption, with space for a free-text response.

## Results

### Categorical comparisons

Categorical comparisons were conducted with independent samples *t*-tests. For the seven exploratory analyses, a Bonferroni correction was applied by multiplying the estimated *p* value by seven. Those values are reported below as adjusted *p* values.

H_1_ posited that the clinical group would show higher levels of insomnia, greater fear of sleep, higher scores on the psychopathology dimensions, and greater nap desire. We tested these hypotheses using simple *t*-tests and one-way ANOVA. Results are shown in Table 1 and reflect that the clinical sample scored higher than the non-clinical sample in anxiety (8.63 ± 5.76 vs. 3.99 ± 4.82, *t*(653.89) = −11.51, *p* < 0.001, Cohen’s *d* = −0.88 [95% CI -1.03, −0.72]), depression (10.09 ± 6.70 vs. 4.70 ± 5.32, *t*(636.52) = −11.74, *p* < 0.001, Cohen’s *d* = −0.90 [95% CI -1.05, −0.74]), internalizing (3.38 ± 2.32 vs. 1.66 ± 1.98, *t*(660.18) = −10.544, *p* < 0.001, Cohen’s *d* = −0.80 [95% CI -0.96, −0.65]), detachment (3.65 ± 2.62 vs. 1.66 ± 2.10, *t*(639.25) = −11.02, *p* < 0.001, Cohen’s *d* = −0.84 [95% CI -0.99, −0.69]), antagonism (1.16 ± 1.84 vs. 0.62 ± 1.27, *t*(585.96) = −4.49, *p* < 0.001, Cohen’s *d* = −0.34 [95% CI -0.49, −0.20]), disinhibition (2.52 ± 2.09 vs. 1.74 ± 1.83, *t*(667.31) = −5.27, *p* < 0.001, Cohen’s *d* = −0.40 [95% CI -0.55, −0.25]), but not anankastia (3.13 ± 2.44 vs. 3.19 ± 2.46, *t*(697.20) = 0.38, *p* = 0.71, Cohen’s *d* = 0.03 [95% CI -0.12, 0.18]). Clinical participants also reported more insomnia (SCI is scored such that higher numbers reflect better sleep; 15.30 ± 7.85 vs. 21.55 ± 7.77, *t*(703) = 10.61, *p* < 0.001, Cohen’s *d* = 0.80 [95% CI 0.65, 0.95]), greater fear of sleep (12.73 ± 12.74 vs. 5.95 ± 8.46, *t*(571.27) = −8.23, *p* < 0.001, Cohen’s *d* = −0.63 [95% CI -0.78, −0.48]), and were more likely to report wanting to nap (56.12% vs. 45.95%, *F*(1, 703) = 7.34, *p* = 0.007, *φ* = 0.10).

H_2_ through H_4_, which hypothesized that participants with probable insomnia would have higher scores on the internalizing (H_2_), detachment (H_3_), and anankastia (H_4_) scales, were tested via *t*-tests and Cohen’s d, with results reported in Table 2. Those with probable insomnia had higher scores on the internalizing (3.95 ± 2.29 vs. 1.40 ± 1.63, *t*(703) = −17.25, *p* < 0.001, Cohen’s *d* = −1.31 [95% CI -1.48, −1.15]), and detachment scales (4.21 ± 2.55 vs. 1.42 ± 1.82, *t*(703) = −16.97, *p* < 0.001, Cohen’s *d* = −1.29 [95% CI -1.46, −1.13]), but not the anankastia scale (3.06 ± 2.34 vs. 3.23 ± 2.53, *t*(703) = 0.91, *p* = 0.36). Exploratory analyses found that probable insomnia was associated with higher antagonism (1.34 ± 2.04 vs. 0.54 ± 1.03, *t*(703) = −6.86, adjusted *p* < 0.001, Cohen’s *d* = −0.52 [95% CI -0.67, −0.37]) and disinhibition (2.70 ± 2.28 vs. 1.67 ± 1.63, *t*(703) = −6.93, adjusted *p* < 0.001, Cohen’s *d* = −0.53 [95% CI -0.68, −0.38]).

**Table 2:** Psychopathology dimensions and insomnia.

Wright Scale	Normal sleep	Probable insomnia	Significance	Cohen’s *d*	
	M ± SD	M ± SD		Point est.	95% CI
Internalizing	1.40 ± 1.63	3.95 ± 2.29	*t*(703) = −17.25, *p* < 0.001	−1.31	−1.48, −1.15
Detachment	1.42 ± 1.82	4.21 ± 2.55	*t*(703) = −16.97, *p* < 0.001	−1.29	−1.46, −1.13
Antagonism	0.54 ± 1.03	1.34 ± 2.04	*t*(703) = −6.86, *p* < 0.001	−0.52	−0.67, −0.37
Disinhibition	1.67 ± 1.63	2.70 ± 2.28	*t*(703) = −6.93, *p* < 0.001	−0.53	−0.68, −0.38
Anankastia	3.23 ± 2.53	3.06 ± 2.34	*t*(703) = 0.91, *p* = 0.36	0.07	−0.08, 0.22

Participants who reported wanting to nap had higher scores on disinhibition (2.44 ± 2.03 vs. 1.76 ± 1.91, *t*(703) = −4.61, *p* < 0.001, Cohen’s *d* = −0.35 [95% CI -0.50, −0.20]). Exploratory analyses found that nap desire was also associated with higher scores on the internalizing (2.94 ± 2.31 vs. 2.00 ± 2.21, *t*(703) = −5.54, adjusted *p* < 0.001, Cohen’s *d* = −0.42 [95% CI -0.57, −0.27]), detachment (3.08 ± 2.64 vs. 2.12 ± 2.38, *t*(699.31) = −5.04, adjusted *p* < 0.001, Cohen’s *d* = −0.38 [95% CI -0.53, −0.23]), antagonism (1.04 ± 1.60 vs. 0.72 ± 1.56, *t*(703) = −2.65, adjusted *p* = 0.029, Cohen’s *d* = −0.20 [95% CI -0.35, −0.05]) and anankastia scales (3.42 ± 2.51 vs. 2.89 ± 2.36, *t*(702.37) = −2.88, adjusted *p* = 0.058, Cohen’s *d* = −0.22 [95% CI -0.37, −0.07]).

### Profile analyses

Profile analyses were conducted to compare the pattern of scores across the five psychopathology domains (internalizing, detachment, antagonism, disinhibition, anankastia) between two groups (insomnia and nap desire in separate analyses). These analyses were used to determine (a) if the score patterns between the two groups are parallel, which determines if the shape of the score patterns vary by group, regardless of whether one group has a higher or lower overall level; (b) whether one group has consistently higher or lower scores than the other; and (c) whether the subscales differ from each other when both groups are considered. The flatness test (c) was not relevant to the hypotheses and is therefore not discussed.

These analyses were estimated using a multivariate analysis of variance (MANOVA) with structured contrasts (Morrison, 2005; Tabachnick and Fidell, 2022), estimated via the repeated-measures general linear model in SPSS. Thus subscale was a five-level within-subject factor and group was the between-subject factor. Mauchly’s test was used to assess sphericity, and Greenhouse–Geisser-corrected degrees of freedom were reported when the sphericity assumption was violated.

Models were estimated in simplified form, without covariates, and then run again with covariates to determine if the inclusion of demographic and/or other relevant covariates meaningfully changed the results. Depression and anxiety symptoms were not included as covariates because the Wright et al. (2025) psychopathology scales are intended to operationalize broad transdiagnostic dimensions of psychopathology that conceptually overlap with traditional symptom measures such as the PHQ-9 and the GAD-7. Statistically adjusting for these measures would therefore remove variance that the Wright scales were specifically designed to capture, effectively shifting the research question from characterizing psychopathology profiles associated with sleep disturbance to examining residual variance unique to one measurement framework while accounting for another (see Miller and Chapman, 2001). Evaluation of the incremental validity of these alternative measurement approaches is an important question but was beyond the scope of the present study.

#### Profile analysis: insomnia

**Figure 1:**
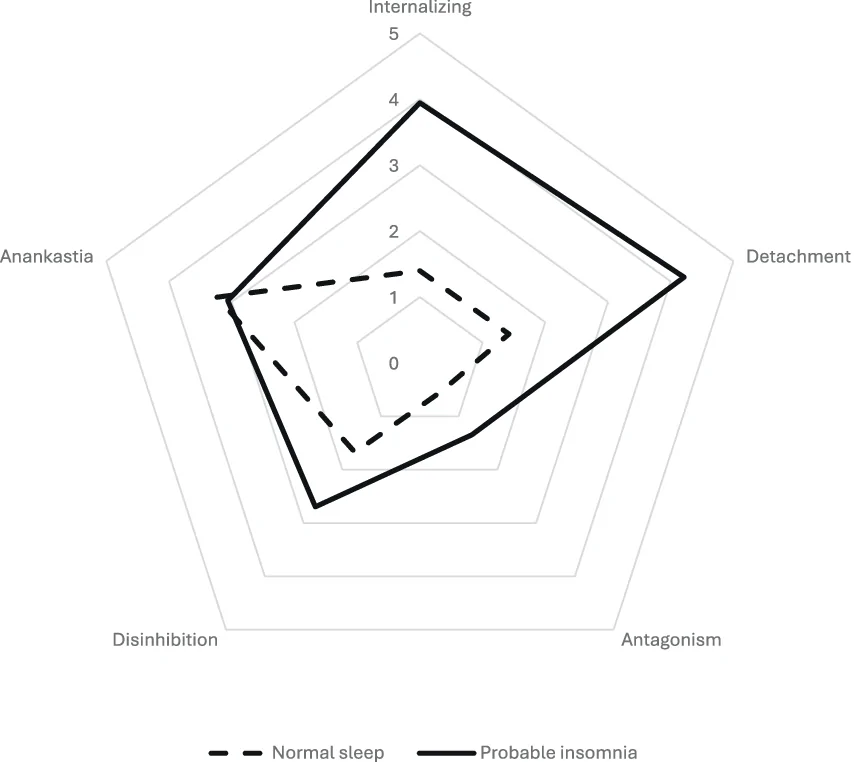
Profile of dimensions of psychopathology scale scores for probable insomnia and normal sleep.

**Figure 2:**
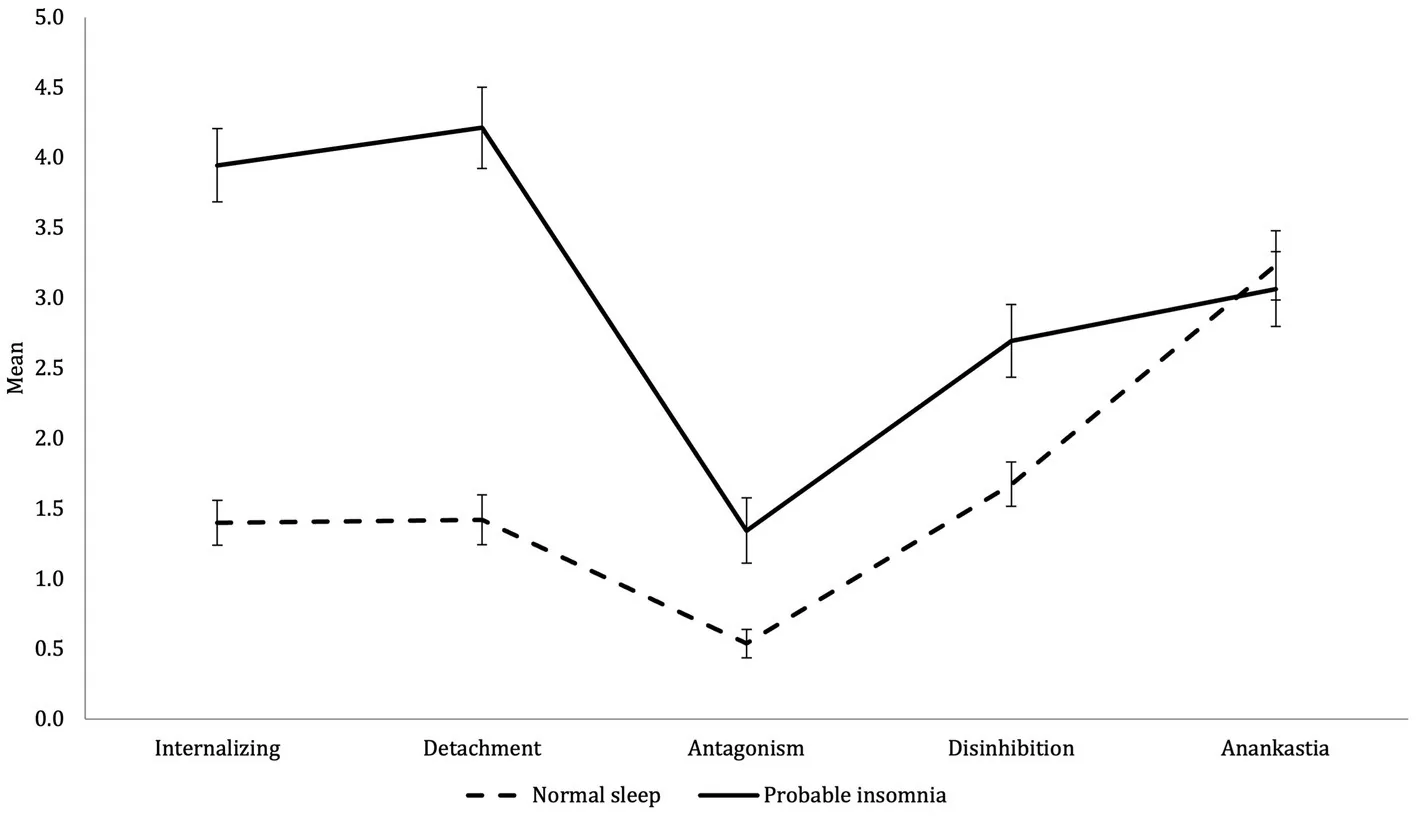
Line plot of dimensions of psychopathology scale scores for participants with probable insomnia and normal sleep. Error bars represent 95% CI.

A profile analysis was conducted using SPSS, implemented as a multivariate analysis of variance (MANOVA) with structured contrasts to test the parallelism and level hypotheses. This analysis was used to determine if the pattern of scores on the five psychopathology dimensions (internalizing, detachment, antagonism, disinhibition, and anankastia) was significantly different for those with probable insomnia (based on SCI scores). Mauchly’s test indicated that the assumption of sphericity was violated, W = 0.512, χ^2^(9) = 469.79, *p* < 0.001; Greenhouse–Geisser *ε* = 0.729. A main effect of probable insomnia was observed, *F*(1, 703) = 178.57, *p* < 0.001, partial η^2^ = 0.20 [95% CI 0.15, 0.25], suggesting that participants with probable insomnia exhibited generally higher scores on the five psychopathology dimensions compared to those who did not. More importantly, however, the subscale by probable insomnia interaction was significant, *F*(2.92, 2049.56) = 96.52, *p* < 0.001, partial η^2^ = 0.12 [95% CI 0.10, 0.15], suggesting a distinct profile of scores associated with probable insomnia (see Figures 1, 2). Independent *t*-tests indicated that participants with probable insomnia scored higher on internalizing, detachment, disinhibition, and antagonism, but not anankastia (see Table 2).

##### Sensitivity

A model in which insomnia was measured by the SCI total score as a continuous variable (as opposed to the dichotomized version, above) was estimated. The results of this model did not vary in sign or significance, and the observed effect increased slightly in magnitude. Since both the subscale by SCI interaction, *F*(2.94, 2063.38) = 115.15, *p* < 0.001, partial η^2^ = 0.14 [95% CI 0.11, 0.17], and the main effect of SCI, *F*(1, 703) = 278.09, *p* < 0.001, partial η^2^ = 0.28 [95% CI 0.23, 0.33], replicate the dichotomized version, Table 2 and Figures 1, 2 show only the dichotomized version for ease of interpretation.

##### Covariates

The model was estimated with demographic, nap, and demographic and nap covariates. The results did not vary in direction or significance. In the first, demographic covariates (age, SES, dummy-coded gender, clinical status) were added; in the second, nap desire and nap behavior were added, and in the third, demographic and nap covariates were added. The direction and significance of results did not vary; the interaction and main effects remained significant in all models, though demographic covariates attenuated the effect sizes. In the adjusted model, the subscale × probable insomnia interaction remained significant, *F*(3.07, 2134.51) = 41.59, *p* < 0.001, partial η^2^ = 0.06 [95% CI 0.04, 0.08] (vs. 0.12 unadjusted), as did the main effect of probable insomnia, *F*(1, 696) = 105.31, *p* < 0.001, partial η^2^ = 0.13 [95% CI 0.09, 0.18] (vs. 0.20 unadjusted). Attenuation was attributable almost entirely to the demographic covariates; nap variables produced negligible change alone or incrementally. For ease of interpretation, the model described in Table 2 and Figures 1, 2 is the model without covariates.

#### Profile analysis: nap desire

**Figure 3:**
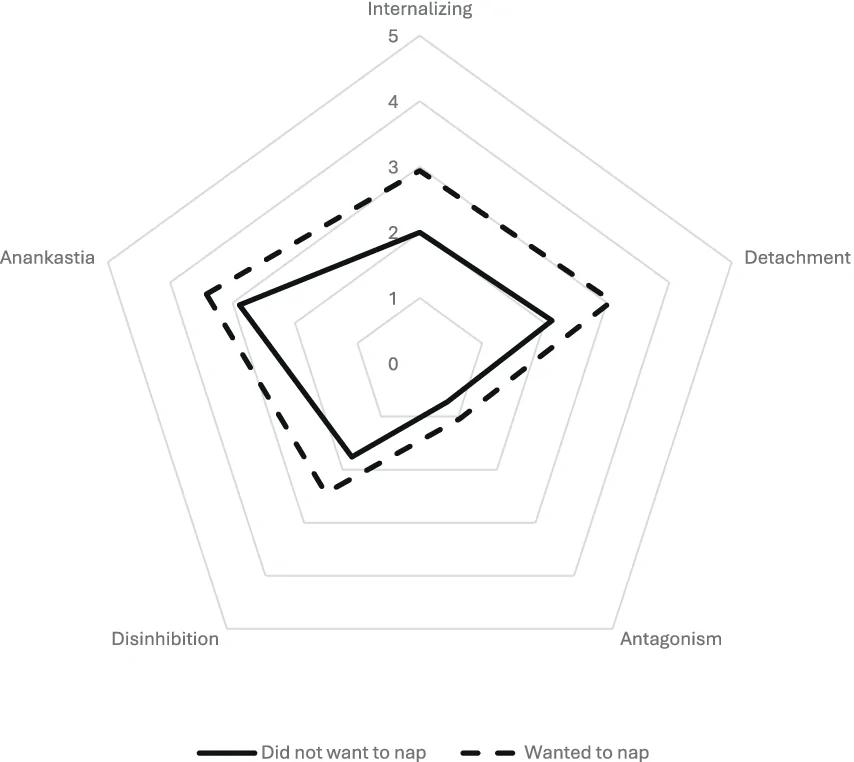
Profile of dimensions of psychopathology scale scores for participants with nap desire and those without nap desire.

**Figure 4:**
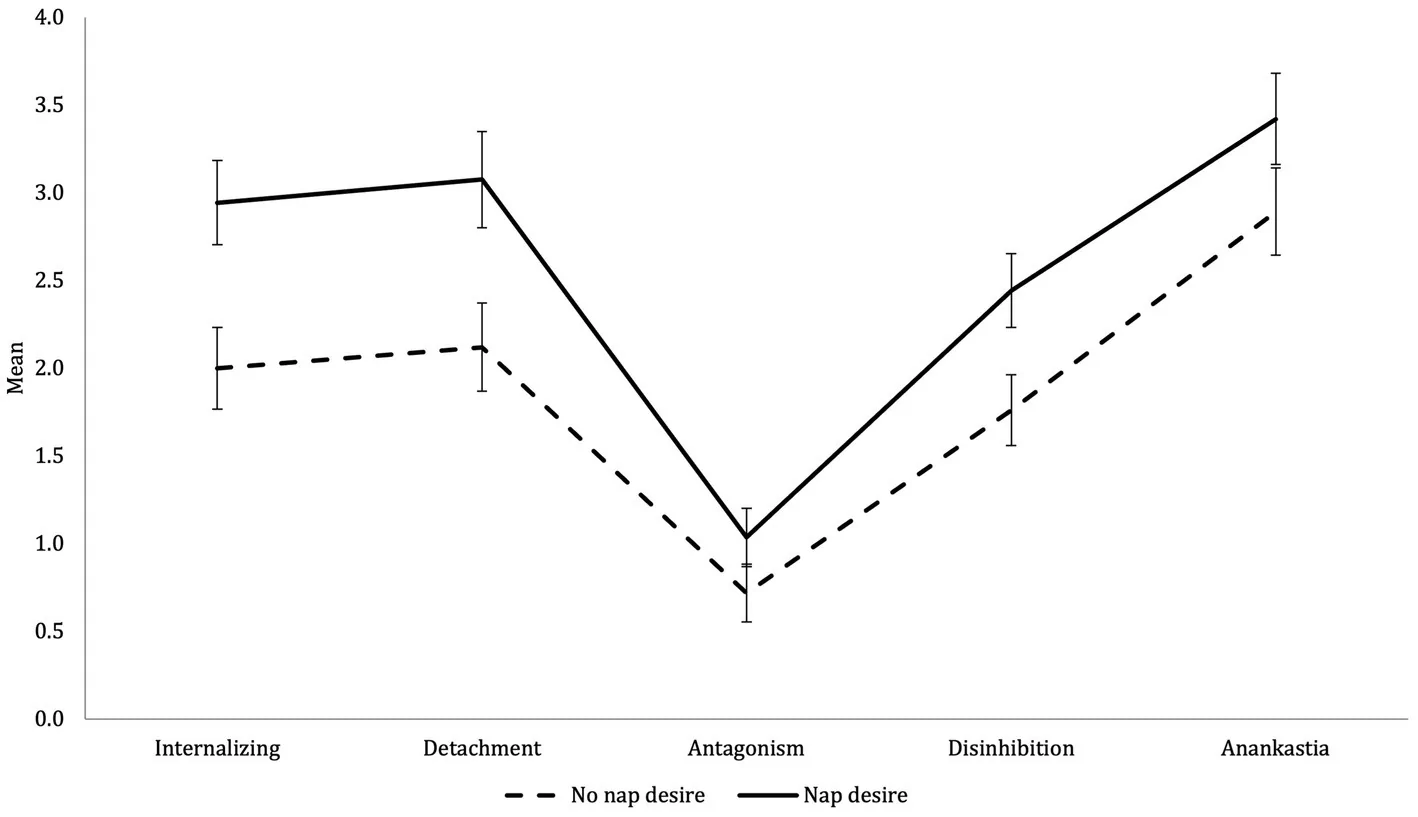
Line plot of dimensions of psychopathology scale scores for participants with nap desire and those without nap desire. Error bars represent 95% CI.

A profile analysis was conducted using SPSS to determine if the pattern of scores on the five psychopathology dimensions (internalizing, detachment, antagonism, disinhibition, and anankastia) was significantly different for those who indicated they wanted to nap today (nap desire) vs. those who did not want to nap (no nap desire). Mauchly’s test indicated that the assumption of sphericity was violated, W = 0.416, χ^2^(9) = 614.64, *p* < 0.001; Greenhouse–Geisser *ε* = 0.667. A main effect of nap desire was observed, *F*(1, 703) = 36.78, *p* < 0.001, partial η^2^ = 0.05 [95% CI 0.02, 0.08]. The subscale by nap desire interaction was significant, *F*(2.67, 1876.71) = 4.22, *p* = 0.008, but had a small effect size, η^2^ = 0.006 [95% CI 0.0005, 0.014], suggesting a distinct profile of scores associated with wanting to nap, but with relatively small differences (see Figures 3, 4). Independent *t*-tests indicated that participants who wanted to take a nap today scored higher on all dimensions in psychopathology scales than those who did not want to take a nap (see Table 3).

**Table 3:** Psychopathology dimensions and nap desire.

Wright Scale	Did not want to nap	Wanted to nap	Significance	Cohen’s *d*	
	M ± SD	M ± SD		Point est.	95% CI
Internalizing	2.00 ± 2.21	2.94 ± 2.31	*t*(703) = −5.54, *p* < 0.001	−0.42	−0.57, −0.27
Detachment	2.12 ± 2.38	3.08 ± 2.64	*t*(699.31) = −5.04, *p* < 0.001	−0.38	−0.53, −0.23
Antagonism	0.72 ± 1.56	1.04 ± 1.60	*t*(703) = −2.65, *p* = 0.008	−0.20	−0.35, −0.05
Disinhibition	1.76 ± 1.91	2.44 ± 2.03	*t*(703) = −4.61, *p* < 0.001	−0.35	−0.50, −0.20
Anankastia	2.89 ± 2.36	3.42 ± 2.51	*t*(702.37) = −2.88, *p* = 0.004	−0.22	−0.37, −0.07

##### Covariates

Just as with the previous profile analysis, this model was run with the addition of key demographic variables (age, SES, dummy-coded gender, clinical status). As with the previous analysis, the direction and significance of the results did not vary. The main effect of nap desire remained significant, *F*(1,698) = 29.63, *p* < 0.001, partial η^2^ = 0.04 [95% CI 0.02, 0.07], as did the interaction, *F*(2.98, 2078.56) = 3.26, *p* = 0.021, partial η^2^ = 0.005 [95% CI 0.0001, 0.011]. For ease of interpretation, the model described in Table 3 and Figures 3, 4 show the model without covariates.

## Discussion

The present study examined how insomnia and nap desire, two common but distinct sleep-related experiences, correspond to broad dimensions of psychopathology. Our hypotheses were largely supported. Our first hypothesis was that these data would replicate and extend existing findings such that those in the clinical sample would have more insomnia and fear of sleep, as well as be more likely to report wanting to nap, and score higher on all of the dimensions of psychopathology subscales compared to participants in the non-clinical sample. This hypothesis was supported, confirming both that sleep challenges are related to general psychopathology, and that the abbreviated dimensions of psychopathology are effective at distinguishing between a clinical and non-clinical sample.

Hypotheses two, three and four tested links between sleep condition and three specific dimensions of psychopathology: internalizing, detachment, and anankastia. These hypotheses were partially supported, with higher scores in internalizing and detachment observed, but not in anankastia. In exploratory analyses, sleep impairment was associated with higher scores on antagonism and disinhibition. These results support both measurement and clinical conclusions, including that the dimensions of psychopathology as measured by the scales from Wright et al. (2025) differ by sleep status, but not uniformly. The lack of an association with anankastia warrants careful interpretation. Although cognitive models of insomnia frequently emphasize perfectionistic tendencies, rigid sleep-related beliefs, and pre-sleep rumination as maintaining factors, the broad anankastia spectrum assessed by the Wright et al. (2025) scales extend beyond these sleep-specific cognitive processes to encompass more general rigidity, compulsivity, and restraint. Thus, our findings should not be interpreted as evidence that perfectionism is unrelated to insomnia. Rather, they may suggest that insomnia aligns more closely with internalizing and detachment at the spectrum level, or alternatively that more narrowly defined sleep-related perfectionistic cognitions are not fully captured by the abbreviated anankastia measure. Future studies using more comprehensive HiTOP (Kotov et al., 2017) and perfectionism measures will be important for clarifying these relationships.

Nap desire was found to be associated with higher levels of disinhibition. Although prior work has linked daytime sleepiness and sleep disruption to disinhibited behavior (Schmidt et al., 2010; Winsper and Tang, 2014; Bauducco et al., 2019; Kakoschke et al., 2025; Rossa et al., 2014), these findings extend this literature by suggesting that subjective desire to nap may also be associated with disinhibitory psychopathology. While nap desire and daytime sleepiness are distinct constructs, one of many likely predictors of nap desire is daytime sleepiness (other predictors may include low motivation or low mood) (Duggan et al., 2016). Importantly, however, nap desire was found to be related to higher scores on all the psychopathology dimensions, suggesting that napping desire may be associated with psychopathology on multiple levels, either via connection to a p-factor (Caspi et al., 2014) or another cross-dimensional, transdiagnostic mechanism like avoidance (Campbell et al., 2022; Zanfirescu Șerban et al., 2025).

Finally, hypotheses five and six, which posited the existence of distinct profiles of transdiagnostic scales for insomnia and nap desire, were supported. Consistent with a large body of literature linking sleep disturbance with mental health difficulties (Baglioni et al., 2016; Freeman et al., 2020; Pandi-Perumal and Kramer, 2010), participants with probable insomnia reported both a different profile of psychopathological scales and significantly higher levels of psychopathology across most dimensions, particularly internalizing, detachment, disinhibition, and antagonism. This pattern is broadly consistent with dimensional models such as HiTOP (Kotov et al., 2017), in which insomnia appears to align most strongly with the internalizing spectrum while also showing meaningful associations with adjacent domains such as detachment and aspects of externalizing psychopathology. Nap desire was also associated with elevated scores across all dimensions, though effect sizes were smaller and less differentiated than those observed for insomnia. Taken together, these findings suggest that insomnia and nap desire may represent partially overlapping but distinct markers of vulnerability to psychopathology.

Results suggest that insomnia may be associated with a more differentiated psychopathology profile, while nap desire corresponds to a more generalized elevation across domains. Specifically, insomnia was most strongly linked with internalizing and detachment, indicating a potential relationship between chronic difficulties initiating or maintaining sleep and affective distress combined with social withdrawal. Although the present study cannot address underlying mechanisms, these associations are broadly consistent with neurobiological models suggesting that chronic insomnia is characterized by persistent hyperarousal and alterations in neural systems involved in emotion regulation, salience detection, and reward processing (Riemann et al., 2015). Such mechanisms may contribute to both heightened negative affect (internalizing) and reduced motivation for or engagement in social interactions (detachment), although longitudinal and mechanistic studies are needed to test these hypotheses directly. Elevated antagonism and disinhibition also point to associations with irritability, hostility, and impulsive behavior, consistent with prior evidence that sleep disturbance contributes to emotion regulation difficulties and externalizing risk (Harvey, 2008; Liu et al., 2022; Palmer and Alfano, 2017). By contrast, nap desire was linked to modest but consistent elevations across all dimensions, with slightly less specificity. This pattern suggests that a persistent wish to nap may function more as a general marker of psychological distress, aligning with prior work demonstrating that nap desire is a stronger predictor of depressed and anxious mood than actual napping (Ostrow et al., 2024). Hypersomnolence and excessive sleepiness have likewise been associated with diffuse psychological impairment (Saad et al., 2023; Soehner et al., 2014).

From a clinical perspective, these findings underscore the importance of considering sleep-related processes when assessing transdiagnostic psychopathology. The links between insomnia and both internalizing and detachment suggest that these domains frequently co-occur and may warrant concurrent clinical assessment. Whether interventions targeting these dimensions improve sleep or broader mental health outcomes remains an important question for future longitudinal and intervention studies. Cognitive Behavioral Therapy for Insomnia (CBT-I) is well-established as an effective intervention (Trauer et al., 2015), and studies are needed to determine whether improvements in insomnia are accompanied by changes in broader psychopathology dimensions, including interpersonal detachment and antagonism. While the clinical implications of nap desire are less well established, our results suggest that persistent or frequent desire to nap could be associated with multiple domains of psychopathology, warranting further assessment. Importantly, nap desire should not be interpreted as synonymous with pathological daytime sleepiness, but rather as a subjective experience that may reflect multiple psychological and physiological processes. Incorporating brief assessments of nap desire into routine clinical evaluations may provide a low-burden way to identify individuals who are experiencing diffuse psychological distress. Given the relatively small effect sizes observed, we hope that future research can replicate and refine these findings.

The current study also has broader implications for dimensional approaches to sleep research. By applying the Wright et al. (2025) scales, we demonstrated that sleep disturbances map onto distinctive profiles of transdiagnostic psychopathology dimensions, offering a more nuanced characterization than categorical diagnoses alone. While this cross-sectional work can only suggest mechanistic relationships, with future longitudinal work, dimensional mapping may help clarify heterogeneity in sleep–psychopathology associations and guide the development of personalized interventions that target the specific mechanisms most relevant to an individual’s symptom profile. Future work should replicate and extend this approach using the within-person longitudinal design used in Wright et al.’s analysis (Wright et al., 2025). Combined with these results, a longitudinal analysis could establish whether the presence of these distinct profiles predicts differential treatment response or long-term outcomes, whether dimensional psychopathology profiles predict differential treatment response, and whether interventions that explicitly target dimensions such as detachment or antagonism yield additional benefits beyond existing symptom-focused treatments. A within-person longitudinal analysis may also be able to speak even more clearly to the effect of the problematic emotion regulation approach that we speculatively describe as napping- or sleeping-as-avoidance. An additional direction for future research will be to directly compare dimensional psychopathology frameworks, such as the Wright et al. scales, with more traditional symptom measures of depression and anxiety. Although these approaches reflect different conceptualizations of psychopathology rather than simple covariates, future studies should examine the extent to which dimensional psychopathology profiles provide complementary or incremental explanatory and predictive value for sleep disturbances beyond conventional symptom measures. Such work would help clarify when dimensional approaches offer unique clinical or scientific advantages for understanding the relationships between sleep and psychopathology.

Several limitations should be noted when interpreting these findings. The cross-sectional design precludes inferences about causal relationships between sleep disturbances and psychopathology dimensions; longitudinal studies are needed to clarify temporal dynamics and directionality. In addition, all measures relied on self-report, which may be influenced by recall bias or subjective interpretation, particularly for constructs such as nap desire and clinical status. While third-party confirmation of psychiatric diagnosis is untenable in a large online survey, the absence of that validation weakens the distinctions between the clinical and non-clinical groups, and readers are advised to interpret those distinctions with caution, absent later replication. Incorporating objective measures of sleep and circadian functioning (e.g., actigraphy, polysomnography) and ecological momentary assessment of daily experiences would strengthen future research, as would assessment of chronotype preference and medication effects. The use of a single-day assessment of nap desire/behavior (e.g., “Did you take a nap today?”) adds clarity and reduces the likelihood of biased or inaccurate memory, but it also reduces the window of assessment significantly. The number of people reporting napping today is thus very likely to be a minimum and not an exact estimate. Recruiting the sample online may have introduced selection bias and, while stratified into clinical and non-clinical groups, may not generalize to more diverse populations or to those with severe psychiatric conditions who are less likely to participate in online studies. Although the Wright et al. (2025) scales demonstrated good psychometric properties in this sample, their original development focused on within-person variability rather than cross-sectional comparisons, and additional validation work is warranted. Finally, while our analyses accounted for attention checks, replication across independent samples will be important to confirm the robustness of these findings.

Despite these limitations, this study demonstrates that insomnia and nap desire correspond to distinct yet overlapping profiles of transdiagnostic psychopathology dimensions, highlighting the utility of dimensional frameworks in clarifying heterogeneity in sleep-mental health associations. Clinically, if replicated, these findings support the routine assessment of both insomnia and nap desire as potential indicators of psychopathology. They also suggest that dimensions such as detachment and disinhibition may represent promising candidates for future intervention research examining whether combining evidence-based sleep interventions, like CBT-I, with strategies targeting these dimensions may enhance prevention and treatment. By situating sleep disturbances within transdiagnostic models of psychopathology, this work contributes to a growing effort to integrate sleep health into transdiagnostic, mechanism-driven mental health interventions.

## Data Availability

The raw data supporting the conclusions of this article will be made available by the authors, without undue reservation.
